# Fracture behavior of short fiber-reinforced CAD/CAM inlay restorations after cyclic fatigue aging

**DOI:** 10.1007/s10266-023-00815-y

**Published:** 2023-04-25

**Authors:** S. Garoushi, D. Barlas, P. K. Vallittu, M. B. Uctasli, L. Lassila

**Affiliations:** 1 Department of Biomaterials Science and Turku Clinical Biomaterial Center–TCBC, Institute of Dentistry, University of Turku, Turku, Finland; 2 Department of Restorative Dentistry, Faculty of Dentistry, University of Gazi, Ankara, Turkey; 3 Wellbeing Services County of South-West Finland, Turku, Finland

**Keywords:** CAD/CAM, Fatigue aging, Inlays, Short fiber composite, Fracture behavior

## Abstract

The aim of this study was to assess the fracture behavior of molar teeth restored with MOD inlays made of experimental short fiber-reinforced CAD/CAM composite block (SFRC CAD) before and after cyclic fatigue aging. Standardized MOD cavities were prepared on 60 intact mandibular molars. Three groups of CAD/CAM made inlay restorations (Cerasmart 270, Enamic, and SFRC CAD) were fabricated (*n* = 20/group). All restorations were luted with self-adhesive dual-cure resin cement (G-Cem One). Half of restored teeth per each group (*n* = 10) were quasi-statically loaded until fracture without aging. The other half underwent cyclic fatigue aging for 500,000 cycles (*F*_max_ = 150 N) before being loaded quasi-statically until fracture. Then, the fracture type was visually inspected. The microstructure and elemental content of CAD/CAM materials were assessed using SEM and EDS. Two-way analysis of variance (ANOVA) was used to statistically examine the data, and it was followed by the Tukey HSD test (*α* = 0.05). ANOVA demonstrated that both material type and aging had a significant effect (*p* < 0.05) on the load-bearing capacity values of the restorations. Teeth restored with SFRC CAD showed significantly the highest (*p* < 0.05) load-bearing capacity (2535 ± 830 N) after fatigue aging among all groups. SEM images showed the ability of short fibers in SFRC CAD composite to redirect and hinder crack propagation. With regard to fracture mode, Enamic group revealed 85% of catastrophic failure (vs. 45% and 10% for Cerasmart 270 and SFRC CAD, respectively). Large MOD cavities on molar teeth were most favorably restored with SFRC CAD inlays, yielding the highest load-bearing capacity and more restorable failures.

## Introduction

Restoring posterior teeth with significant coronal damage is a challenge that arises often in clinical practice. With the recent advances in the adhesive dentistry, several minimally invasive treatment approaches are available. Preserving the remaining tooth structure is essential to maintain the restoration and is seen as an important factor when selecting alternative approaches. Modern fabrication methods for indirect restorations have advanced concurrently with modern materials. The use of computer-aided design and manufacturing (CAD/CAM) technology has replaced the conventional hand-layering method [1]. However, there is still concern about the clinical durability of indirect inlay/onlay restoration, particularly for posterior teeth with significant tooth structure loss. Fracture of restoration and debonding were shown to be the most frequent causes of failure [2]. Notably, in the case of MOD (mesio-occluso-distal) cavities, studies from the literature revealed that loss of both marginal ridges and relative cuspal stiffness resulted in a 54% reduction in the fracture strength of teeth [3, 4]. In addition, one author stated that under occlusal load, the cusp that remains after MOD cavity preparation behaves as a cantilever beam: the cavity's floor functions as a fulcrum for cusp bending, and the cantilever's length rises as the cavity's depth [5]. Consequently, restorative technique and material selection of these MOD cavities require careful consideration [6, 7].

Lithium disilicate glass ceramic has been employed for definitive prosthesis in general as a material for indirect bonded restorations due to its superior mechanical characteristics. It includes a significant number of longitudinal crystals, which improves its bond strength, fracture resistance, and flexural strength [8, 9]. However, lithium disilicate glass ceramic is a stiff and brittle material that requires further crystallization to reach its full strength and has been observed to induce wear to antagonist dental enamel [10]. Nevertheless, because of the high elastic modulus, it has a tendency to concentrate additional stress at the adhesive interface, which could result in adhesion failure [11]. Accordingly, some researchers promote the use of materials with similar elastic modulus to dentin to obtain a more reasonable stress distribution. Composite CAD/CAM materials have gained popularity during the last decade, because they demonstrate favorable elastic modulus, wear properties, color integration, and millability in thin layers [12, 13]. Because it was polymerized at a high temperature and pressure, it has much more monomer conversion than conventionally light-cured composites, which results in composites with greater mechanical and biological properties. Additionally, polymerization shrinkage of restorations made of this material is restricted to the cement space. However, the efficiency of machinable composites remains controversial as their mechanical properties can be shown to be inferior to those of ceramics [14].

To enhance the mechanical properties of conventional particulate-filled composites and reduce issues that could adversely affect the long-term clinical success, short fiber-reinforced composites (SFRCs) have been developed [15, 16]. Recently, experimental SFRC CAD/CAM composite was introduced with the aim of improving the fracture toughness of conventional particulate-filled CAD/CAM composites. Studies demonstrated promising performance when mechanical, optical, surface, and bonding properties were tested [17–20]. Consequently, there is no information on the fracture behavior of this material when used to construct intracoronal inlays. Thus, the current work aimed to compare the fracture behavior of MOD inlays constructed of SFRC CAD/CAM composites to commercially available hybrid CAD/CAM composites before and after cyclic fatigue aging. It was hypothesized that the tested restorations' fracture behavior is unaffected by the aging process or the material type.

## Materials and methods

Table 1 includes a list of the materials utilized in this study along with their composition.

**Table 1:** CAD/CAM materials used in the study

Materials	Manufacturers	Composition according to manufacturer
Cerasmart 270	GC Corp, Tokyo, Japan	Bis-MEPP, UDMA, dimethacrylate, Silica (20 nm), barium glass (300 nm) 71 wt%
Enamic	VITA Zahnfabrik, Bad Säckingen, Germany	UDMA, TEGDMA, glass ceramic 86 wt%
SFRC CAD	Experimental	UDMA, TEGDMA, Short glass fiber (200–300 µm & Ø7 μm), Barium glass 77 wt%

*TEGDMA* triethylene glycol dimethacrylate, *UDMA* urethane dimethacrylate, *Bis-MEPP* Bis (p-methacryloxy (ethoxy) 1–2 phenyl)-propane, *wt%* weight percentage

Sixty extracted mandibular molar teeth with similar occlusal size (± 1 mm) and free of caries were utilized. Following collection, attached soft tissues were washed away with running water, and then, the teeth were kept at 4 °C in a 0.5% chloramine T solution for a maximum of 2 months. Using a digital caliper (Mitutoyo Corp., Tokyo, Japan), the size of each tooth was measured from different directions. The mean dimensions were 10.3 (± 0.5) in bucco-lingual and 11.3 (± 0.6) in mesio-distal directions. Thereafter, the teeth were mounted from the cement-enamel junction (CEJ) on an acrylic block (diameter 2.5 cm) using auto-polymerized acrylic resin (Palapress; Heraus Kulzer, Wehrheim, Germany). A similar coronal preparation was performed on all teeth. Preparations and restorations of teeth were done by a single operator.

### Tooth preparation and restorative procedures

**Fig. 1:**
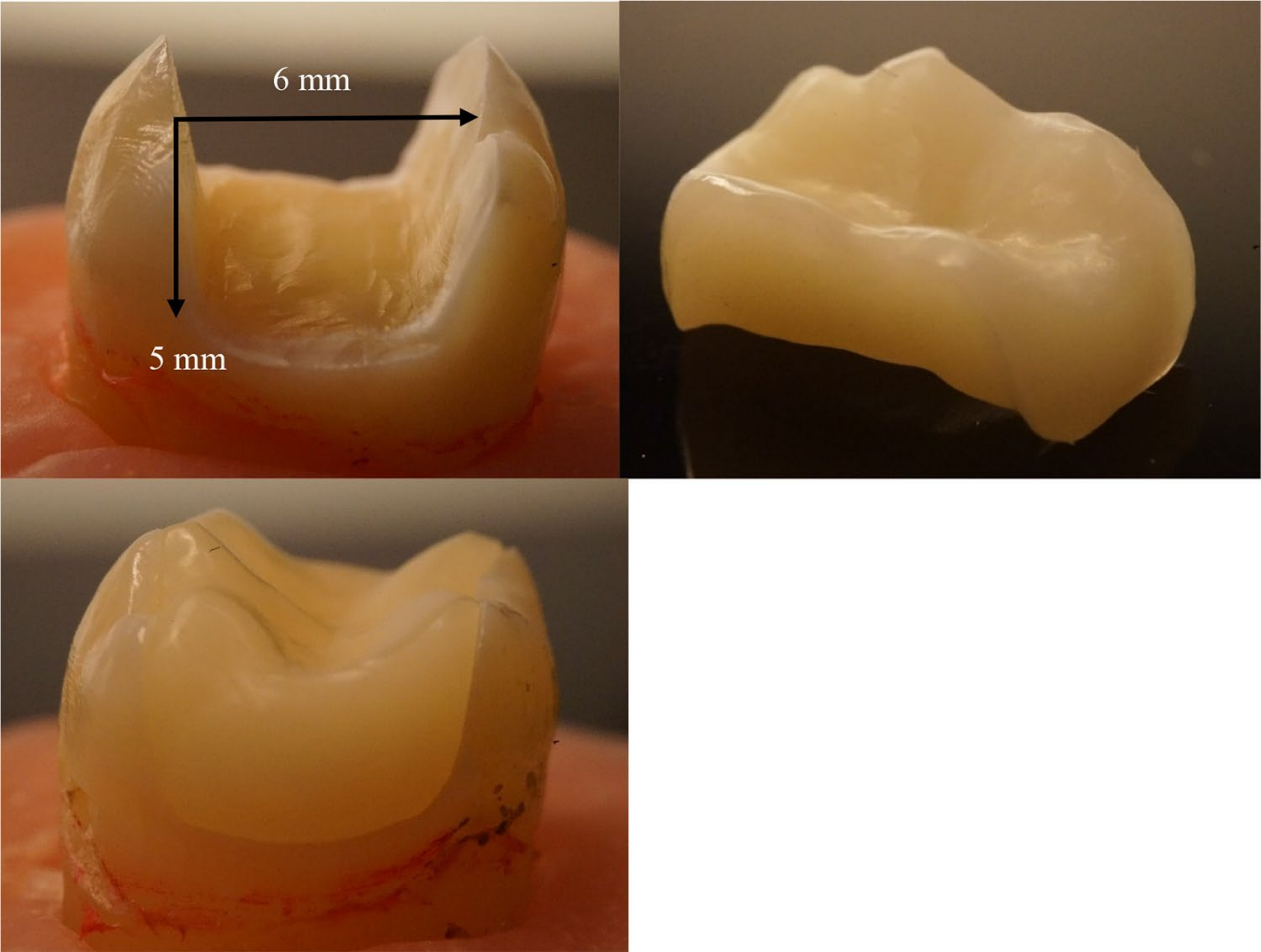
Photographs show the standard MOD tooth preparation with corresponding measurements (5 mm in depth, 6 mm in bucco-palatal width) and test specimen

Standard preparations simulated a large MOD cavity preparations were fabricated using high-speed handpiece with flat-end parallel carbide (H21LR.314.010, Brasseler, Savannah, GA, USA) and round-end diamond burs (850–014 M SSWhite, Lakewood, NJ, USA) under water cooling. The preparation was made having a flat cavity floor with a depth of 5 mm and 6 mm in bucco-palatal width (Fig. 1). The buccal wall's remaining thickness was about 2 mm (digital caliper, Mitutoyo Corp., Kawasaki, Japan). The margins were positioned 1–2 mm above CEJ. After completing the cavity preparation, the tooth surfaces were prepared for bonding with a selective acid-etching (37% phosphoric acid) and with adhesive using one-bottle universal bonding agent (G-Premio Bond, GC Corp., Tokyo, Japan) according to the manufacturers’ instructions. The teeth were then randomly distributed in three groups.

After taking a digital photoimpression (dental intraoral scanner; CEREC) of the prepared cavity, restoration was designed and milled (CEREC, Sirona Dental Systems Inc., Long Island City, NY) using Cerasmart 270, Enamic and SFRC CAD blocks. All restorations had their inner surfaces acid etched with 4.5% hydrofluoric acid (IPS Ceramic Etching Gel, Ivoclar, Schaan, Liechtenstein) for 60 s prior to cementation (according to the manufacturer’s instructions). This was followed by washing and air-drying. The restorations were then luted using a multi primer (G-Multi Primer, GC, Tokyo, Japan) and self-adhesive dual-cure resin cement (G-Cem One, GC, Tokyo, Japan). After that, light curing from all directions for 20 s per segment using a hand-light curing unit (Elipar TM S10). The inlay surface was in close proximity to the light source. All restorations underwent polishing using abrasive polishing tips (Jiffy Polishers, Ultradent, South Jordan, UT, USA) and were then kept in water for 2 days at 37 °C before testing.

### Fracture load test

Prior to the quasi-static fracture load test, cyclic fatigue aging was applied to half of the restored teeth per group (*n* = 10). The specimens were placed in a 37 °C water bath in a chewing simulator (MOD, Esetron Smart Robotechnologies, Ankara, Turkey). Then, specimens were subjected to 500,000 cycles of mechanical dynamic loading at a frequency of 1.5 Hz and a force of Fmax = 150 N. Following cyclic fatigue aging, a quasi-static load was directly subjected to the other half of the specimens in each group (*n* = 10/group) using a universal testing machine (Lloyd model LRX, Lloyd Instruments Ltd, Fareham, UK) at a speed of 1 mm/min. Using a metal ball (5 mm in diameter), loading was delivered vertically between the lingual and buccal cusps' triangular ridges. The loading curve was observed up until the restoration fractured (last incline in the load–deflection curve).

### Fracture mode and microstructure analysis

Three independent examiners agreed on the failure type, position, and direction after visually examining the specimen's fracture type. For fractographic evaluation, representative fractured specimens were examined using scanning electron microscopy (SEM) (LEO, Oberkochen, Germany). The analysis started from the edge of the fractured specimens from the upper loading surfaces to the inner structures.

SEM and energy-dispersive spectroscopy (EDS) provided the characterization of the microstructure of the investigated CAD/CAM materials. Prior to observation, all specimens were coated with a gold layer using a vacuum evaporator and a sputter coater (BAL-TEC SCD 050 Sputter Coater, Balzers, Liechtenstein).

### Statistical analysis

The data were statistically analyzed with SPSS version 23 (SPSS, IBM Corp.) using a two-way analysis of variance (ANOVA) at the *p* < 0.05 significance level, followed by a Tukey HSD post hoc test to determine the differences between the groups. The independent factors were the material type and aging process, and the dependent variables were the values of the fracture load. To examine whether results showed typical variation, Levene's test was applied.

## Results

**Fig. 2:**
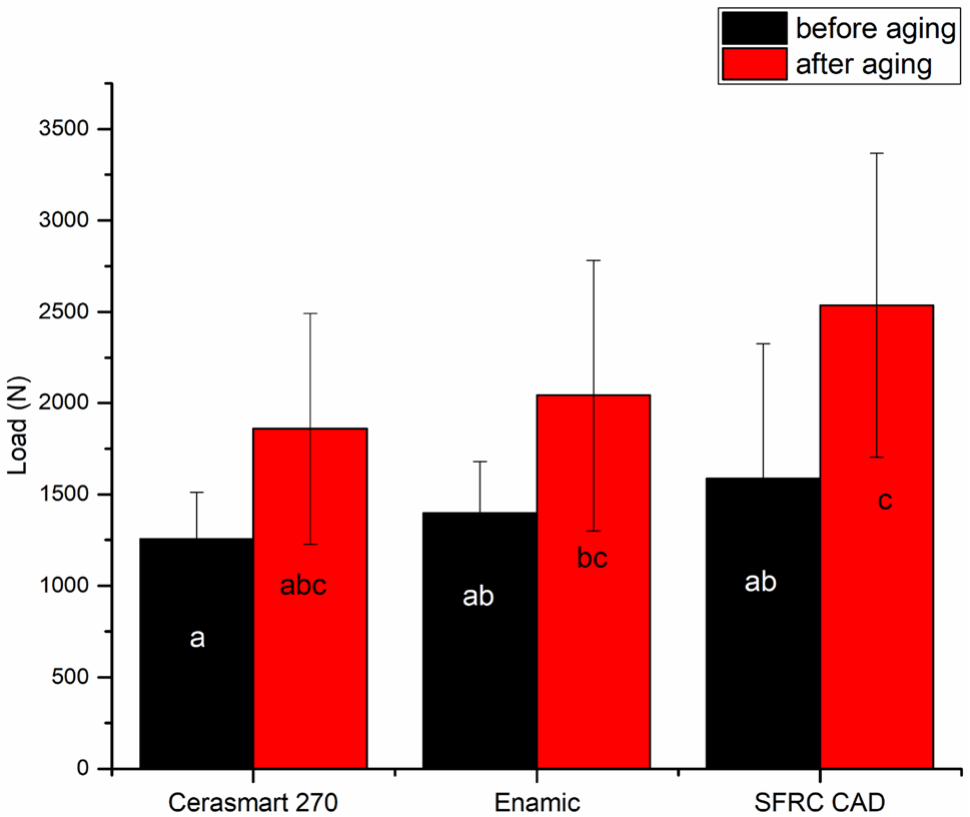
Mean values of fracture load (N) and standard deviation (SD) of tested restorations before and after cyclic fatigue aging. The same letters inside the bars represent non-statistically significant differences (*p* > 0.05) among the materials

Throughout the 500,000 cyclic fatigue aging, none of the restored teeth experienced failure. Therefore, static loading to failure was applied to all of the fatigued restorations. The load-bearing capacity of the tested restorations was significantly affected by both material type and aging technique (*p* < 0.05), according to two-way ANOVA analyses. Figure 2 displays the average load-bearing capacity values with standard deviations of the tested restorations both before and after cyclic fatigue aging. The variances were homogeneous and equal among groups, according to Levene's test. The experimental SFRC CAD restorations exhibited the highest load-bearing capacities among the tested groups only after aging. However, before aging, there was no significant difference (*p* > 0.05) between the SFRC CAD and Enamic groups. The data revealed that after cyclic fatigue aging, the load-bearing capacity values of all tested restorations significantly increased (*p* < 0.05).

Despite the application of substantial loading forces, none of the restorations failed adhesively. Visual inspection of the specimens revealed three different fracture types (Table 2). Chipping of only tooth structure (Type 1) occurred predominantly in the SFRC CAD specimens (75%). Fracture involving both the restoration and tooth structure (Type II) which was uncommon but mostly observed in Cerasmart 270 specimens (35%). Crushing fracture extending to root (Type III) occurred predominantly in Enamic specimens (85%). This type is a catastrophic fracture that cannot be repaired, where the tooth is completely crushed.

**Table 2:**
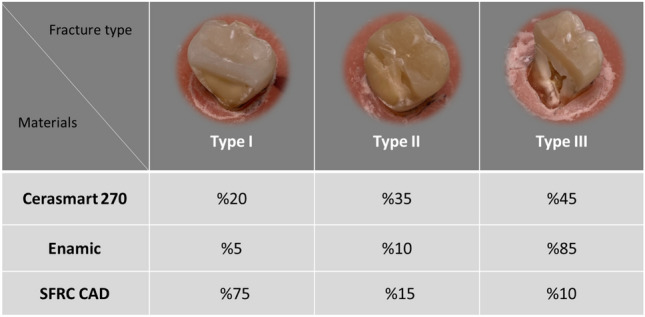
Percentage and photographs of various fracture types of the restorations

*Type I* chipping of only tooth structure, *Type II* fracture involving both the restoration and tooth structure, *Type III* catastrophic fracture extending to root

**Fig. 3:**
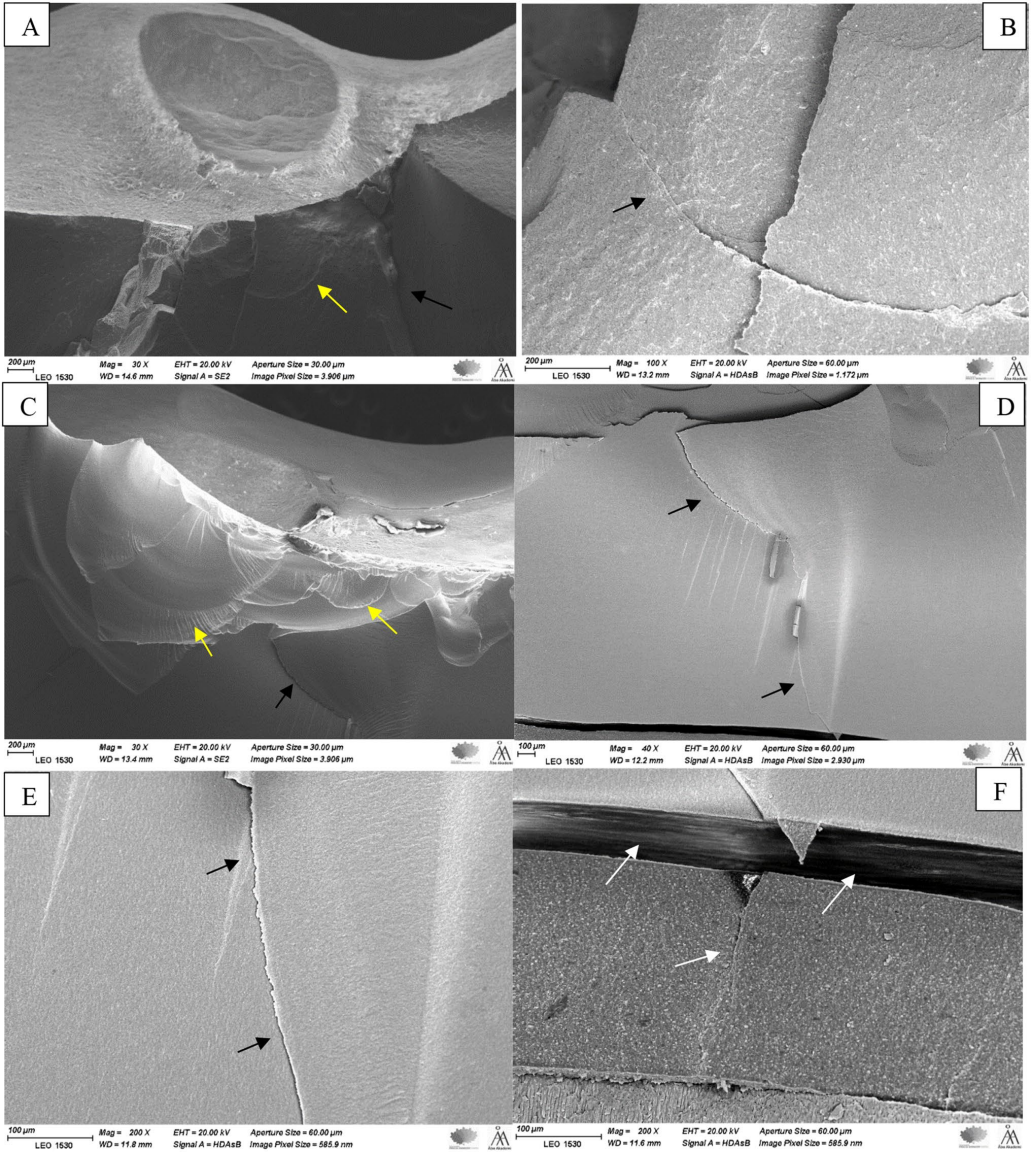
SEM images of the fractured hybrid CAD/CAM composite specimens (Enamic **A** & **B** and Cerasmart 270 **C**–**F**) observed under different magnifications showing arrest lines with twist hackles (**A** & **C**, yellow arrows) and a radial cracks (black arrows) propagated through the restoration from the load application area to the interface with luting cement. Crack and gap at the adhesive interface between restoration and luting cement (**F**, white arrows)

**Fig. 4:**
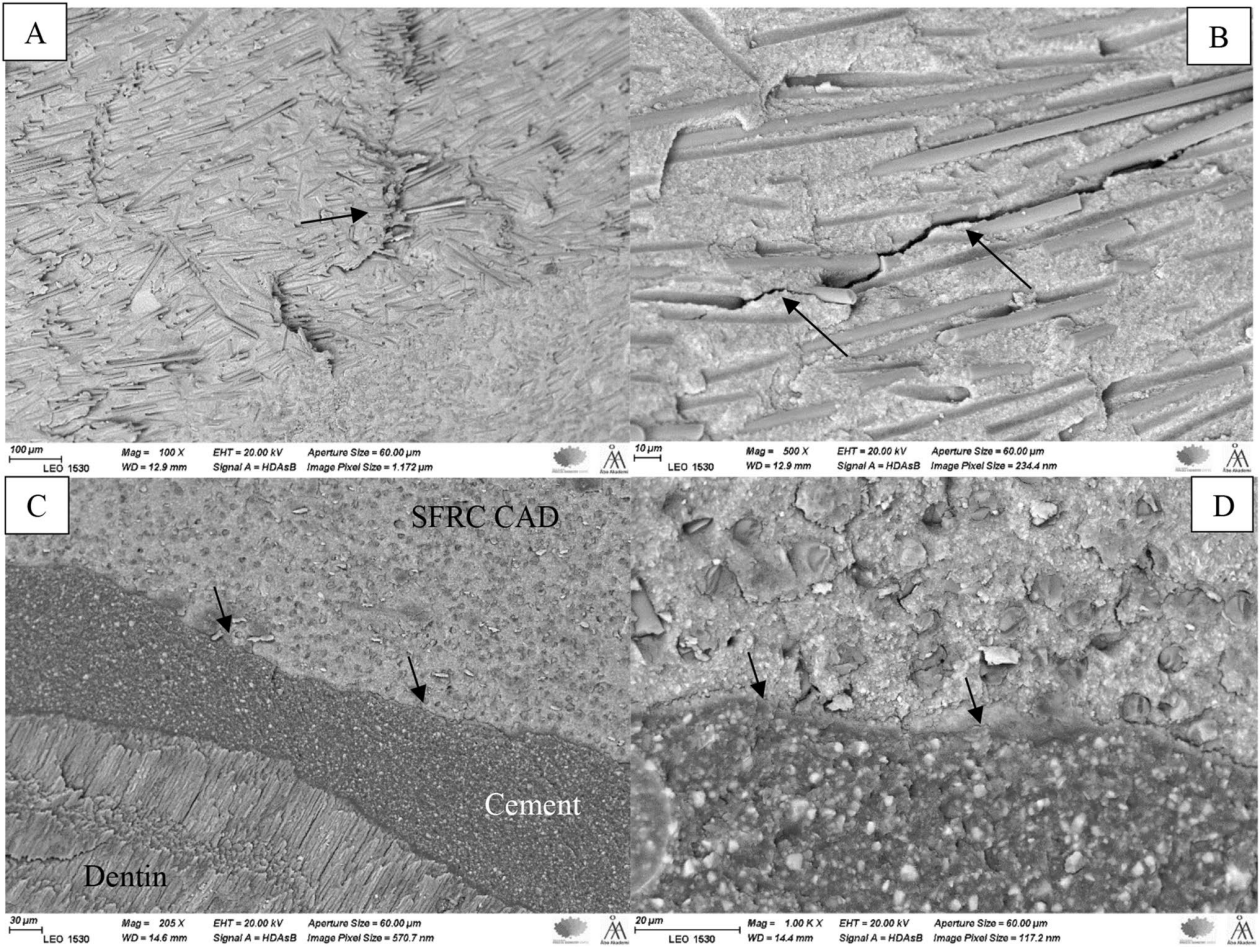
SEM images of the fractured fiber-reinforced CAD/CAM composite specimen (SFRC CAD) observed under different magnifications showing short fibers' ability to redirect and hinder crack propagation (**A**, **B**, arrows). **C** & **D** showing the adhesive interface between restoration and luting cement (arrows)

Figure 3 shows the SEM images of the fractured part of hybrid CAD/CAM composite specimens (Enamic & Cerasmart 270). The images (different magnifications) display fracture markers, including arrest lines—multiple concave lines that indicate the crack's radial downward propagation. The images also reveal fine twist hackles starting between the arrest lines. Moreover, a gap was detected at bonding interface between restorations and luting cement. On the other hand, images of an experimental SFRC CAD specimen (Fig. 4, different magnifications) demonstrate how a crack line initially spread before being blocked by fibers. In addition, no gap was detected between restorations and luting cement (Fig. 4).

**Fig. 5:**
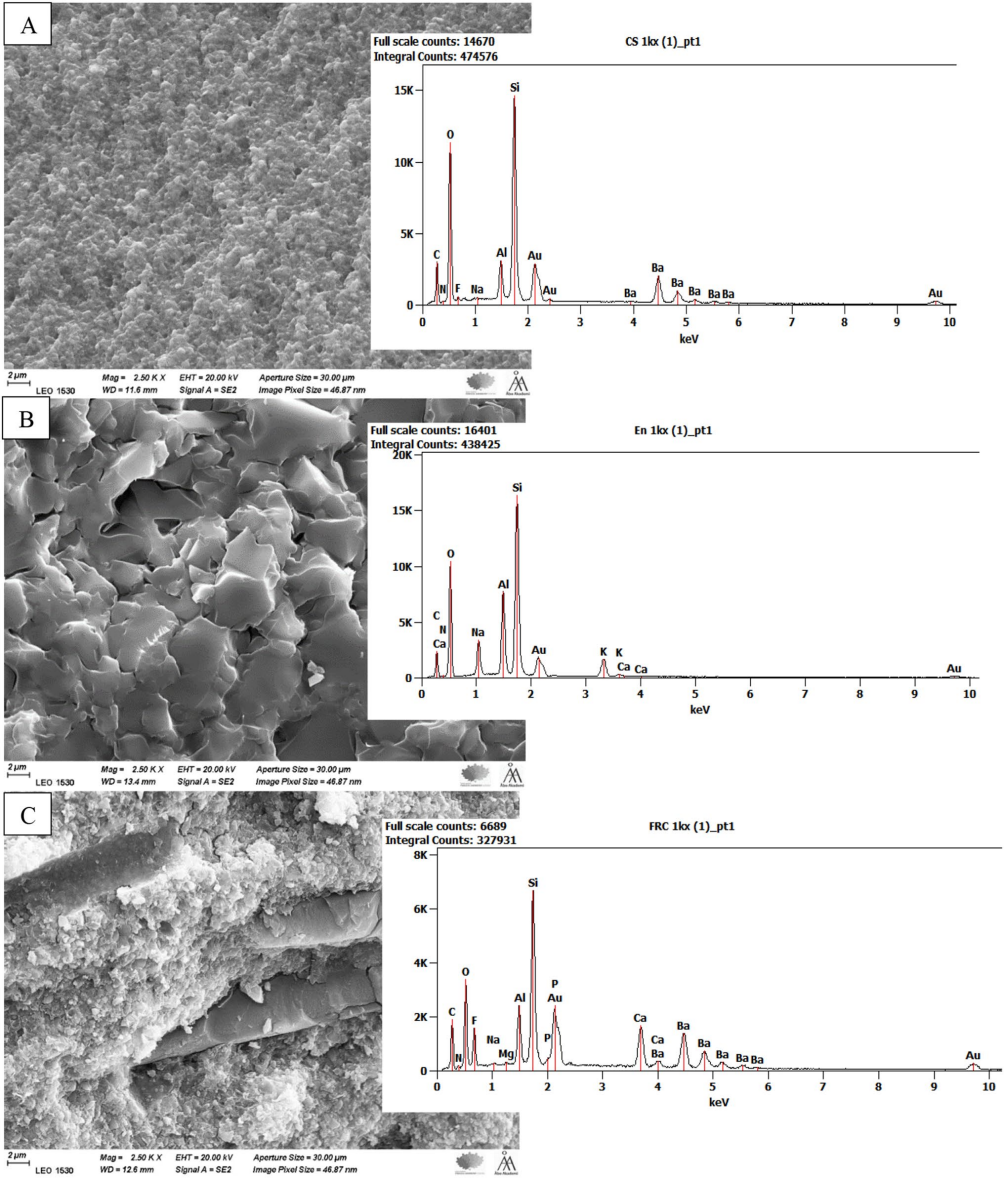
SEM images with EDS analysis of fracture surface of investigated CAD/CAM materials (scale bar = 2 µm). **A** Cerasmart 270; **B** Enamic; **C** SFRC CAD

SEM/EDS analysis (Fig. 5) presented the microstructure of each tested material with different compositions, from particulate nano-filled polymer matrix (Cerasmart 270) to sintered ceramic matrix with some polymer network (Enamic), to short fiber and particulate-reinforced polymer matrix (SFRC CAD). This proposed an explanation for the varying performance between the tested materials.

## Discussion

The present study showed that material type and aging had a considerable effect on the fracture behavior of the tested restorations; therefore, the study hypothesis was rejected. CAD/CAM hybrid materials can be divided into two categories based on their microstructure (Fig. 5): resins with scattered particulate-reinforced fillers and polymer-infiltrated ceramic networks (PICN). Cerasmart 270 is typical example of resins with dispersed particulate-reinforced fillers and PICN is represented by Enamic [21]. On the other hand, experimental SFRC CAD is composite reinforced with discontinuous glass fibers. It is comprised of a resin matrix, glass microfibers that are oriented randomly, and inorganic particle fillers (Fig. 5C).

The findings of the present investigation demonstrate that the experimental SFRC CAD inlays had the highest load-bearing capacity values. These results are in accordance with the other studies that showed significant improvement in fracture behavior of SFRC CAD in comparison with different composite and ceramic materials [18, 20, 22]. According to the literature, there are two explanations for this finding. First, higher load-bearing capacity values can result from stress transferring from the polymer matrix to the glass fibers. Second, the glass fibers improve the material's fracture behavior by deflecting the cracks individually. This increases the energy required for crack propagation through the polymer matrix [23–25]. In comparison to the experimental SFRC CAD composite restorations, both Cerasmart 270 and Enamic restorations had lower load-bearing capacity values, with a statistically significant difference (*p* < 0.05) after cyclic aging (Fig. 2). This could be explained by the relatively high brittleness and low fracture toughness of Cerasmart 270 and Enamic in comparison to SFRC CAD. Fracture toughness has been defined in the literature as a mechanical parameter that describes brittle materials' resistance to the catastrophic propagation of cracks under an applied force [26, 27]. As a result, it provides information about the material's capacity to withstand damage and acts as a reference for fracture resistance. The reported fracture toughness value of the experimental SFRC CAD was 2.9 MPa m^1/2^ [18], while for Enamic and Cerasmart 270, it was in range between 1.4 and 1.8 MPa m^1/2^ [18, 28]. However, considering that the maximum masticatory forces in the molar region may reach up to 900 N [29], the materials examined in the current study were able to resist fracture loads that were significantly greater than the reported masticatory forces in the molar area. Interestingly, after cyclic fatigue aging, the load-bearing capacity values of all tested restorations were significantly higher than those of the same material before aging (Fig. 2). This is in line with previous studies that found fatigue cracks in composites are much less likely to develop in wet conditions and that the development of fatigue cracks can be slowed down [20, 30, 31]. The specimens were kept in the water path (37 °C) for a number of days during the aging process, which may account for these outcomes. The polymer matrix absorbs water in aqueous conditions that might promote plasticization [30]. Due to the residual compressive stress generation at the fatigue crack tips, this phenomena may result in blunting and decreased stress concentration. In addition, it results in the release of developed tensile stress during polymerization shrinkage. Both of these effects, either collectively or individually, have the potential to slow the propagation of cracks, increasing the material's capacity to withstand more load. However, other research with different results indicated that the aqueous environment weakens the polymer chain and affects the composite's ability to resist crack propagation; hence, the influence of plasticization on the fracture properties of the composites is controversial [32, 33].

Massive loads were applied (far beyond the masticatory forces), but none of the restorations failed adhesively, which would explain the good bond obtained. The combination of micromechanical interlocking (surface roughness produced by acid-etching) and chemical bonding through the usage of the primer can be used to explain the cohesiveness between the luting cement and the CAD/CAM materials.

Visual analysis of the fracture behavior of the experimental SFRC CAD restorations revealed that chipping of only tooth structure was predominant (Table 2). In fractured specimens, clear surface and subsurface damage was present at the occlusal area owing to the loading process. Discontinuous fibers served as stoppers and prevented further crack development by blocking the fracture crack in the experimental SFRC CAD specimens (Fig. 4). Thereby, the fracture type shifted to mainly repairable fractures, compared to the other tested groups.

The fracture type of the Enamic specimens was primarily catastrophic crushing-like fractures (Table 2). This seems to be median-radial cracks propagate from the load contact site through the entire restoration thickness. Enamic has the highest index of brittleness among all the tested materials [28]. The reason may be that Enamic is a hybrid ceramic block material and was made via PICN technology. First, a totally dominant porous ceramic network was built, and then, resin monomers were incorporated. The greater flexural modulus and hardness were caused by the high loading and ceramic skeleton [34]. On the other hand, the lower brittleness of Cerasmart 270 leads to less catastrophic fracture than Enamic restorations [28]. SEM images show a radial distribution of the crack lines propagated from the loading area to the deeper structure. Fractographic markers, such as twist hackle and arrest lines, are clearly displayed in SEM images (Fig. 3). According to Scherrer et al., arrest lines are good signs of the direction of crack propagation, since the start of a crack event typically occurs on the concave side of the initial arrest line [35].

Studies in the literature have shown that luting cements can be subjected to high loading stresses, which can result in luting cement microfracture or cracks that eventually create gap at interface and catastrophic failure [36, 37]. Interestingly, SFRC CAD restorations were tightly connected to the luting cement and dentin, minimizing the drawbacks of using a weak link between them (Fig. 4). This is in accordance with Mangoush et al., who showed that shear-bond strength value of SFRC CAD composite to resin cement was higher than that of conventional hybrid CAD/CAM composite [17].

The findings of this study have to be seen in light of some limitations, absence of thermal aging long-term water storage, and other well-known and proven restorative CAD/CAM materials, such as lithium disilicate or monolithic zirconia. The absence of a periodontal ligament simulation is another limitation of this study. Therefore, further research is needed to gain more knowledge about the experimental SFRC CAD composite.

Despite the stated limitations, the use of both static and dynamic loading setups separately in the current study is a strength from a practical point of view.

## Conclusion

Large MOD cavities on molar teeth were most favorably restored with SFRC CAD inlays, yielding the highest load-bearing capacity and more restorable failures.

## Data Availability

Data available within the article.
